# The Modulation of Apoptotic Pathways by Gammaherpesviruses

**DOI:** 10.3389/fmicb.2016.00585

**Published:** 2016-04-27

**Authors:** Shuvomoy Banerjee, Timsy Uppal, Roxanne Strahan, Prerna Dabral, Subhash C. Verma

**Affiliations:** ^1^Amity Institute of Virology and Immunology, Amity UniversityNoida, India; ^2^Department of Microbiology and Immunology, Center for Molecular Medicine, School of Medicine, University of Nevada, RenoReno, NV, USA

**Keywords:** apoptosis, EBV, KSHV, cancer, autophagy, LANA, EBNA3C

## Abstract

Apoptosis or programmed cell death is a tightly regulated process fundamental for cellular development and elimination of damaged or infected cells during the maintenance of cellular homeostasis. It is also an important cellular defense mechanism against viral invasion. In many instances, abnormal regulation of apoptosis has been associated with a number of diseases, including cancer development. Following infection of host cells, persistent and oncogenic viruses such as the members of the *Gammaherpesvirus family* employ a number of different mechanisms to avoid the host cell’s “burglar” alarm and to alter the extrinsic and intrinsic apoptotic pathways by either deregulating the expressions of cellular signaling genes or by encoding the viral homologs of cellular genes. In this review, we summarize the recent findings on how gammaherpesviruses inhibit cellular apoptosis *via* virus-encoded proteins by mediating modification of numerous signal transduction pathways. We also list the key viral anti-apoptotic proteins that could be exploited as effective targets for novel antiviral therapies in order to stimulate apoptosis in different types of cancer cells.

## Introduction

### Cancer and Apoptosis

Cancer progression can be considered as mechanistically complex process with a plethora of fundamental genetic grounds. Neoplasia, i.e., abnormal growth of cells, involves multiple steps that occur gradually, starting with primary driver mutations and finally leading to tumorigenesis. During these transitional changes, cancer cells accumulate several genetic alterations that confer on the cells, an unwarranted survival and uncontrolled proliferative advantage. During development, cancer cells also encounter a physiologically ubiquitous cellular process, i.e., apoptosis or programmed cell death that eliminates the infected, damaged or abnormal cells. Therefore, it is quintessential for tumor cells to acquire counter-strategies to evade cellular apoptosis that helps to safeguard the genomic integrity (Fernald and Kurokawa, 2013). Defective and inefficient cell death, in turn, facilitates cancer development and metastasis and renders the cancer cells resistant to treatment (Brown and Attardi, 2005).

#### Hallmarks of Apoptosis

##### Morphological hallmarks

The cells undergoing apoptosis display typical morphological and biochemical features. The morphological alterations in apoptosis concerning both the nucleus and the cytoplasm are remarkably similar across various cell types and species (Saraste and Pulkki, 2000). Morphological hallmarks of apoptosis in the nucleus are chromatin condensation, nuclear fragmentation, subsequent rounding up of the cell, pyknosis, and retraction of pseudopodes (Kroemer et al., 2005). The chromatin condensation initiates at the periphery of the nuclear membrane and forms a crescent or ring-like structure and continues until it breaks up inside a cell with an intact membrane, a feature, defined as karyorrhexis (Majno and Joris, 1995). Morphological features for late stage of apoptosis include membrane blebbing, ultrastructural modification of cytoplasmic organelles and a loss of membrane integrity (Kroemer et al., 2005). Several studies demonstrated that proteolytic cleavages of a group of cellular proteins including, actin, spectrin, gelsolin, b-catenin, PAK2, Gas2, MEKK1 by activated caspases play major role for accomplishing the morphological changes during apoptosis (Martin and Green, 1995). Enhanced expression of the caspase cleaved forms of Gas2 or gelsolin were observed for significant change in the cell morphology, resembling apoptosis (Kothakota et al., 1997). Interestingly, experimental evidences showed calpain proteases (Brown et al., 1997) to be associated with the alterations in the cytoskeleton structure during apoptosis (Saraste and Pulkki, 2000).

##### Biochemical hallmarks

Some of the major biochemical changes that can be observed in apoptosis include, activation of caspases, DNA and protein breakdown, membrane changes and recognition by phagocytic cells (Hengartner, 2000). In early stage of apoptosis, the expression of phosphatidylserine (PS) is observed in the outer layers of the cell membrane, which are “flipped out” from the inner layers. This phenomena permits early recognition and phagocytosis of dead cells by macrophages without the release of pro-inflammatory cellular components (Hengartner, 2001). A characteristic breakdown of DNA by endonucleases then results in large 50–300 kb fragments. Inter-nucleosomal cleavage of DNA into oligonucleosomal pieces of 180 to 200 base pairs has been noticed in the later stage of apoptosis (Vaux and Silke, 2003). In particular, generation of free 3′-hydroxyl termini on DNA via cleavage of chromatin into single as well as multiple oligonucleosome-length fragments was considered as one of the major biochemical hallmarks of apoptosis (Loo, 2011). Although this characteristic feature of apoptosis is not very specific as the typical DNA ladder in agarose gel electrophoresis can be observed in case of necrotic cells as well (McCarthy and Evan, 1998). Another striking feature of apoptosis is the activation of a group of enzymes belonging to the cysteine protease family named caspases (Hengartner, 2000). Caspases activation leads to the cleavage of vital cellular proteins and breakdown of the nuclear scaffold and cytoskeleton. Additionally, they activate DNAse, which then promotes the degradation of nuclear DNA (Lavrik I.N. et al., 2005).

### Apoptosis Signal Pathways

It has been well reported that functional activation of caspases play a crucial role in apoptosis in mammalian system (Fritz et al., 2006). Caspases can be activated by either of the two known apoptotic signaling pathways, i.e., intrinsic (mitochondria-mediated) and extrinsic (death receptor-mediated) pathways. Both these pathways ultimately converge to a final common pathway involving the activation of caspases that triggers the execution of apoptosis of the cell. Interestingly, there is a third, less understood intrinsic pathway, referred to as intrinsic endoplasmic reticulum (ER) pathway, which involves ER and is believed to occur in response to cellular stress (Breckenridge et al., 2003).

#### Extrinsic or Receptor-Mediated Pathway

The extrinsic death-receptor pathway is activated upon the death ligands binding with the death receptors (Ozoren and El-Deiry, 2003). Variety of death receptors, such as type 1 TNF receptor (TNFR1) and Fas (CD95) receptor, with their ligands, termed as TNF and Fas ligand (FasL), respectively, participate in the apoptotic pathway (Hengartner, 2001). These death receptors possess an intra-cellular death domain that recruits adapter proteins including, TNF receptor-associated death domain (TRADD), Fas-associated death domain (FADD), and caspase-8 (Schneider and Tschopp, 2000). The death ligand and the death receptor binding results in the formation of a binding site for adaptor protein, and the total ligand-receptor-adaptor protein complex is considered as the death-inducing signaling complex or DISC (Wong, 2011). DISC formation initiates the assembly and activation of pro-caspase 8, which promotes apoptosis by cleaving other downstream or executioner caspases (Kruidering and Evan, 2000). Examples of other best known death receptors are DR3 (APO-3), DR4 (TNF-related apoptosis inducing ligand receptor 1 or TRAIL-1), DR5 (TRAIL-2), DR 6, ectodysplasin A receptor (EDAR), and NGFR (nerve growth factor receptor; Lavrik I. et al., 2005).

#### Intrinsic or Mitochondria-mediated Pathway

The term “Intrinsic pathway” refers to an initiation of the apoptotic pathway within the cell as a result of several internal stimuli, including, genetic damage, oxidative stress, and hypoxia (Wong, 2011). The intrinsic pathway occurs due to the increased mitochondrial permeability and release of pro-apoptotic molecules, such as cytochrome-c into the cytoplasm (Danial and Korsmeyer, 2004). This pathway is also examined by a special group of proteins that belong to the Bcl-2 family, named after the Bcl-2 gene, originally observed at the chromosomal breakpoint of the translocation of chromosome 18–14 in follicular non-Hodgkin lymphoma (Tsujimoto et al., 1984). While the anti-apoptotic group of Bcl-2 proteins (Bcl-2, Bcl-XL, Bcl-W, Bfl-1, and Mcl-1) regulates apoptosis by blocking the mitochondrial release of cytochrome-c, the pro-apoptotic proteins (Bax, Bak, Bad, Bcl-Xs, Bid, Bik, Bim, and Hrk) act by promoting this mitochondrial release of cytochrome-c. The net balance between the pro- and anti-apoptotic proteins actually determines the fate of apoptosis (Reed, 1997). Apoptosis inducing factor (AIF), second mitochondria-derived activator of caspase (Smac), direct IAP Binding protein with Low pI (DIABLO) and Omi/high temperature requirement protein A (HtrA2) are some of the apoptotic factors that are released from the mitochondrial inter-membrane space into the cytoplasm (Kroemer et al., 2007). Cytoplasmic release of cytochrome-c leads to the activation of caspase-3 *via* formation of apoptosome complex, that consists of, cytochrome-c, Apaf-1 and caspase-9 (Kroemer et al., 2007). Moreover, Smac/DIABLO or Omi/HtrA2 stimulates caspase activation by binding to inhibitor of apoptosis proteins (IAPs) which subsequently interferes with the interaction of IAPs and caspase-3 or –9 (LaCasse et al., 2008).

#### Intrinsic Endoplasmic Reticulum-Mediated Pathway

The intrinsic ER pathway is considered as the third pathway for caspase activation and supposed to be involved in caspase-12-dependent and mitochondria-independent manner (Szegezdi et al., 2003). When the ER is damaged by cellular stresses, such as, hypoxia, free radicals or glucose starvation, unfolding of proteins, reduces protein synthesis and an adaptor protein known as TNF receptor associated factor 2 (TRAF2) dissociates from procaspase-12, resulting in the activation of the ER-mediated pathway (Wong, 2011).

#### Final Pathway

Both the intrinsic and extrinsic pathways converge to caspase-3. Thereafter, caspase-3 cleaves the inhibitor of the caspase-activated deoxyribonuclease, which is responsible for the nuclear apoptosis (Ghobrial et al., 2005). Additionally, downstream caspases induce cleavage of protein kinases, cytoskeletal proteins, DNA repair proteins and inhibitory subunits of endonuclease family and are known to influence the cellular cytoskeleton formation, cell-cycle regulation as well as signal transduction pathways which contribute to the typical morphological changes during apoptosis (Ghobrial et al., 2005).

### Deregulation of Apoptosis in Cancer

#### Impaired Death Receptor Signal Transduction

Death receptors and their ligands are the critical players in the extrinsic apoptotic pathways (Plati et al., 2011). These receptors have a death domain to attract several key molecules for inducing death signal. However, the death ligands can also bind to decoy death receptors without these death domain, as a result of which, the signaling complexes fail to initiate the signal transduction (Lavrik I. et al., 2005). Several abnormalities in the death signaling pathways have been identified, including, down-regulation of the receptor or impairment of receptor function and reduced level in the death signal (Wong, 2011). Decreased membrane expression of death receptors and anomalous expression of decoy receptors have also been reported to play a major role for evading death signaling during different malignancies (Fulda, 2010). Several studies have demonstrated that ligand and death receptor expression during different stages of cervical cancer were linked to a discrepancy between apoptosis and cellular proliferation. In particular, studies by Reesink-Peters et al. (2005) demonstrated that loss of Fas and dysregulation of FasL, DR4, DR5, and tumor necrosis factor-related apoptosis-inducing ligand (TRAIL) in the cervical intra-epithelial neoplasia (Parravicini et al., 2000) are thought to be responsible for cervical carcinogenesis (Reesink-Peters et al., 2005).

#### Enhanced Expression of Anti-apoptotic Proteins

The Bcl-2 family of proteins comprises of the pro-apoptotic and anti-apoptotic proteins that play an essential role in the regulation of intrinsic mitochondria-mediated apoptotic pathway (Gross et al., 1999). Interestingly, Bcl-2, encoded by the *Bcl-2* (B-cell lymphoma 2) gene was the first protein of this family to be recognized, more than 20 years ago (Tsujimoto et al., 1984). All the members of the Bcl-2 family proteins are abundantly present on the outer mitochondrial membrane, are dimers in nature and responsible for membrane permeability either in the form of an ion channel or through the formation of pores (Minn et al., 1997).

#### Reduced Expression of Pro-apoptotic Proteins

The group of pro-apoptotic proteins including, Bid, Bim, Puma, Noxa, Bad, Bmf, Hrk, and Bik are restricted to the BH3 domain. Multiple cellular stress responses resulting from DNA damage, growth factor deficiency, and ER stress, can activate the BH3-only proteins. Members of this group such as, Bax, Bak, and Bok/Mtd, contain all four BH3 domains which are also pro-apoptotic (Wong, 2011). When there is a disturbance in the balance of anti-apoptotic and pro-apoptotic members of the Bcl-2 family, apoptotic deregulation is triggered in the affected cells. Studies by Raffo et al. (1995) showed that overexpression of Bcl-2 protects prostate cancer cells from apoptosis (Raffo et al., 1995) whereas, studies by Fulda et al. (2002) demonstrated that increased expression of Bcl-2 proteins lead to inhibition of TRAIL-induced apoptosis in neuroblastoma, glioblastoma, and breast carcinoma cells. Interestingly, overexpression of Bcl-xL has been reported to confer a multi-drug resistance phenotype in tumor cells and prevent them from apoptosis (Minn et al., 1995). Mutations in the *Bax* gene are also very common in case of colorectal carcinogenesis with microsatellite instability. Miquel et al. (2005) concluded that impaired apoptosis resulting from bax (G)8 frameshift mutations are responsible for growing resistance of colorectal cancer cells to anticancer treatments. Moreover, tumor cells of chronic lymphocytic leukaemia (CLL) showed an anti-apoptotic phenotype with high levels of anti-apoptotic Bcl-2 protein and low levels of pro-apoptotic Bax *in vivo*. Cancer progression in CLL is considered to be due to the reduced level of apoptosis rather than enhanced proliferation *in vivo* (Goolsby et al., 2005). Studies by Pepper et al. (1997) demonstrated an increased Bcl-2/Bax ratio in B-lymphocytes in CLL, both in patients and in cultured conditions. The drug induced apoptosis in these B-lymphocytes was found to be inversely related to Bcl-2/Bax ratios (Pepper et al., 1997).

#### Dysregulated p53 Functions

p53 is not only involved in the apoptotic induction but, it is also a vital player in cell-cycle regulation, differentiation, developmental process, gene amplification, DNA recombination, chromosomal segregation, and cellular senescence (Oren and Rotter, 1999). As a result, p53 is called as the “guardian of the genome” (Lane, 1992) and most importantly defects in the p53 tumor suppressor gene have been associated with more than 50% of human malignancies (Sherr and McCormick, 2002). Recent studies by Avery-Kiejda et al. (2011) showed a subset of target genes of p53 involved in apoptosis and cell-cycle regulations are abnormally expressed in melanoma cells, leading to abnormal p53 activity and contributing to cellular proliferation. *In vivo* mice studies using a N-terminal deletion mutant of p53 (Δ122p53) corresponding to Δ133p53 showed decreased survival rate and profound pro-inflammatory phenotype with reduced apoptosis (Slatter et al., 2011). Additionally, it has been observed that silencing of p53 mutant, followed by downregulated expression of p53 mutant resulted in low colony formation in human cancer cells with induced apoptosis (Vikhanskaya et al., 2007). In 1997, two members of p53 family were identified including, p73 (Kaghad et al., 1997) and p63 (Yang et al., 1998). Both p73 and p63 have significant structural similarity with p53 and are involved in a broad spectrum of biological activities (Collavin et al., 2010). Interestingly, several studies have demonstrated that both p63 and p73 are involved in different cellular response to cancer therapy and both of them are required for p53-induced apoptosis, suggesting the functional relationship among p53 family proteins (Chakraborty et al., 2010; Dotsch et al., 2010).

#### Downregulated Caspases Activities

The caspases are classified into two groups: (i) those related to caspase 1 (caspase-4, caspase-5, caspase-13, and caspase-14) are mostly involved in cytokine processing during inflammatory processes, and (ii) those that play vital role in apoptotic process (e.g., caspase-2, caspase-3, caspase-6, caspase-7, caspase-8, caspase-9, and caspase-10). Moreover, the second group can be further divided into initiator caspases, which are primarily responsible for the initiation of the apoptotic pathway (e.g., caspase-2, caspase-8, caspase-9, and caspase-10) and effector caspases (caspase-3, caspase-6, and caspase-7), which are accountable for the actual cleavage of cellular components during apoptosis (Fink and Cookson, 2005). Therefore, lower levels of caspases or deficiency in caspase function may cause reduced apoptosis or carcinogenesis (Wong, 2011). It was reported that down-regulation of caspase-9 is a frequent event in patients with stage II colorectal cancer with poor clinical outcome (Shen et al., 2010). Studies by Devarajan et al. (2002) demonstrated that caspases-3 mRNA levels in samples from breast, ovarian, and cervical tumors were either found undetectable (breast and cervical tumor sample) or significantly reduced (ovarian tumor sample). Fong et al. (2006) observed a down regulation of both capase-8 and caspase-10 in a cDNA differential expression study and suggested that it may contribute to the pathogenesis of chorio-carcinoma.

#### Deregulated Expression of Inhibitor of Apoptosis Proteins (IAPs)

**FIGURE 1:**
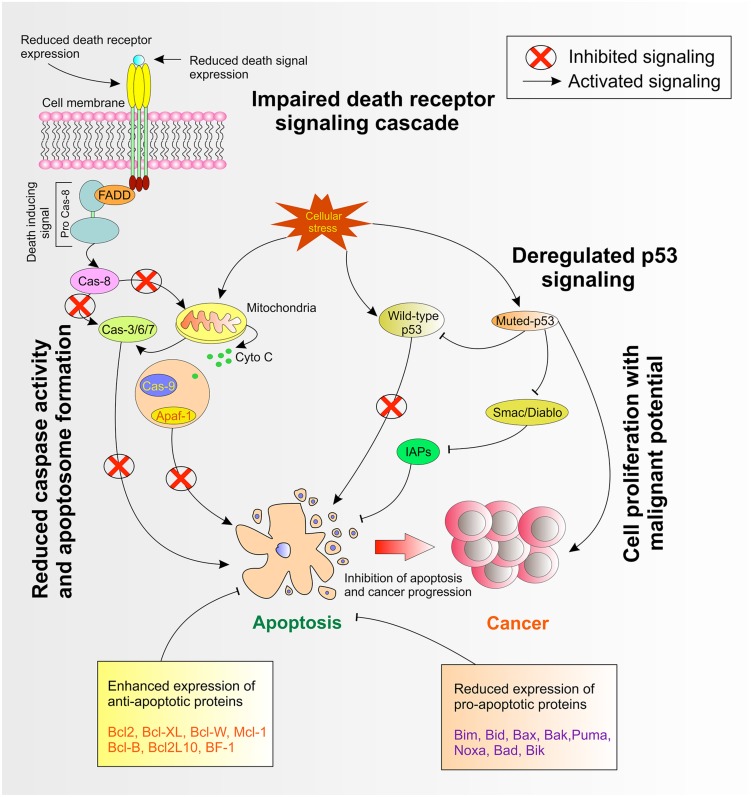
**Deregulated cellular pathways in apoptosis**.

Dysregulated expression of Inhibitor of apoptosis proteins or IAPs has been observed in several cancers (LaCasse et al., 2008). Lopes et al. (2007) observed an irregular expression of the IAP family in pancreatic cancer cells and demonstrated that such expression pattern was also responsible for inducing resistance to cancer chemotherapy. The study concluded that substantial drug resistance correlated with the expression of cIAP-2 in pancreatic cells (Lopes et al., 2007). On the contrary, studies demonstrated that higher IAPs expression are associated with melanoma, lymphoma and gliomas (Ashhab et al., 2001) and was responsible for cisplatin and camptothecin resistance (Chen et al., 1999). Survivin, as potential IAP, is overexpressed in several cancers (Pennati et al., 2007). Interestingly, Small et al. (2010) observed that overexpressed survivin in hematopoietic cells were at an increased risk of hematological malignancies with less susceptibility to apoptosis in transgenic mice (Small et al., 2010). Survivin, together with XIAP is overexpressed in non-small cell lung carcinomas and these tumors were endowed with resistance against different apoptosis-inducing conditions (Krepela et al., 2009). A schematic of cellular pathways involved in apoptosis are shown in **Figure 1**.

### Virus and Cancer

Over past 30 years, it has become remarkably apparent that several viruses play vital roles in the process of carcinogenesis. It is an estimated fact that 15–20% of all human malignancies are associated with viral infections (McLaughlin-Drubin and Munger, 2008). Oncogenic viruses can replicate inside the host cell without being detected by the host’s immune surveillance system and by preventing apoptosis they can protect the cells from self-destruction (Ewald, 2010). Therefore, viruses can be essential experimental models to understand the regulation of major tumor suppressors, identification of major signaling cascades for genome maintenance, apoptotic response, and immune surveillance. Human tumor viruses either belong to RNA virus families including, Retroviridae, Flaviviridae or to DNA virus families such as-Hepadnaviridae, Herpesviridae, and Papillomaviridae. Major tumor viruses, which are linked with a wide range of human malignancies, are HTLV-1, HPV, HHV-8, EBV, HBV, and HCV. Some other viruses that play crucial roles in cancer progression are simian vacuolating virus 40, BK virus, JC virus, human endogenous retroviruses, human mammary tumor virus, Torque teno virus (McLaughlin-Drubin and Munger, 2008). Marek’s disease virus or MDV (also known as Gallid herpesvirus 2) is considered as an α-herpesvirus, which causes oncogenic disease in domestic fowl (chickens) by producing T cell lymphosarcoma (lymphoma), visceral tumors and other clinical signs such as nerve lesions and immunosuppression (Rong et al., 2014). Gallid herpesvirus-2 (GaHV-2) genome integrates into the host genome by homologous recombination and induces transformation of latently infected cells, by modulating the expression of several viral and cellular genes (Gennart et al., 2015).

### Gammaherpesvirus and Cancers

**FIGURE 2:**
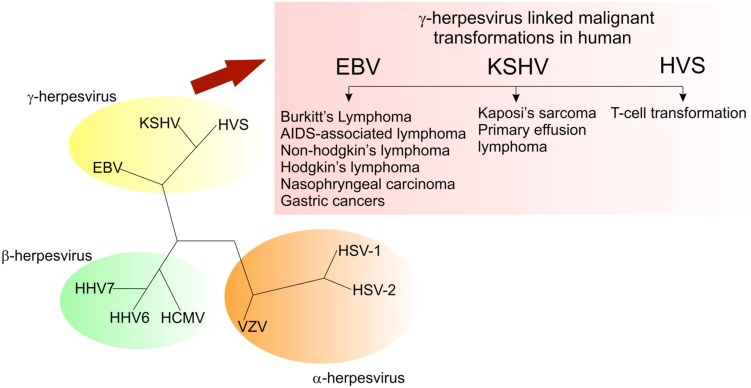
**Members of the gammaherpesvirus family have the ability to drive cell proliferation and tumorigenesis**.

Members of the *Herpesviridae* family, Herpesviruses, are large, double-stranded DNA viruses with a genome size of 100-200 kb, broad species tropism, and known to replicate in the nucleus of the host cell. These viruses are ubiquitous throughout the animal kingdom and are considered as the contributors to lymphomagenesis in immunodeficient humans. Based on their genomic organization, genome sequence and biological characteristics, herpesviruses are sub-classified as α-, β-, and γ-herpesviruses. The γ-herpesviruses are lymphotropic and some of these viruses are capable of undergoing lytic replication in epithelial cells (Klein, 1972). These tumor viruses establish a lifelong latency in the infected host. Interestingly, the γ-herpesviruses show similar genome organization as compared to the members of α- or β-subfamilies (Damania, 2004). The γ-herpesviruses are further divided into two genera: Lymphocryptoviruses (gamma-1 herpesviruses) and Rhadinoviruses (gamma-2 herpesviruses). Lymphocryptoviruses have been identified in higher primates and include *Epstein–Barr virus* (EBV) or Human herpesvirus 4 (HHV-4), whereas the rhadinoviruses are present in a wide range of mammalian species and these include *Herpesvirus saimiri* (HVS), *Kaposi’s sarcoma-associated herpesvirus* (KSHV), or Human herpesvirus 8 (HHV-8), *Rhesus macaque rhadinovirus* (RRV), *Equine herpesvirus 2* (EHV-2), and *Murine gammaherpesvirus 68* (MHV-68). Nearly, all the members of the γ-herpesvirus family share a common property, i.e., the ability to induce neoplasia in natural or experimental hosts (**Figure 2**). Among these γ-herpesviruses, EBV and KSHV are two oncogenic viruses that are linked to the development of multiple human malignancies in their natural hosts. EBV has been linked with several human malignancies, including, Burkitt’s lymphoma, nasopharyngeal carcinoma (NPC), Hodgkin’s disease and a subset of gastric cancers (Blacklow et al., 1971; Ablashi et al., 1985; Mueller, 1991; Magrath, 1992; Magrath et al., 1992; Damania et al., 2000a; Iizasa et al., 2012). Similarly, KSHV has been linked to Kaposi’s sarcoma (KS), multicentric Castleman’s disease (MCD), primary effusion lymphoma (PEL) and more recently, KSHV-associated Inflammatory Cytokine Syndrome (KICS; Chang et al., 1994; Cesarman et al., 1995; Soulier et al., 1995; Gessain et al., 1996; Uldrick et al., 2010; Polizzotto et al., 2012).

#### Lymphocryptovirus

Lymphocryptoviruses or LCVs are considered as gamma 1-herpesviruses which are well known for infecting old-world primates (Dunkel et al., 1972). They exhibit a biphasic life cycle with a lifelong persistence and are capable of promoting malignancies (Cho et al., 1999). As a well-studied LCV, Epstein–Barr virus (EBV) was found only member known to cause infection in humans (Yajima et al., 2008).

##### Role of *Epstein–Barr virus* (EBV) antigens in cancer progression by modulation of apoptotic signaling

*EBNA1.* Previous studies suggested that direct elimination of EBV from Burkitt’s lymphoma (BL) cells induces apoptotic event (Kennedy et al., 2003). Although, most of the experimental evidences suggested that EBV infection mainly responsible for inhibiting apoptosis in cellular transformation but in some cases, EBV infection induces apoptotic response in human neutrophils and may represent an alternative mechanism by which EBV suppresses the immune response (Larochelle et al., 1998). Also, other report showed that EBV could inhibit cord blood monocytes derived dendritic cells (CBDC) phenotypic differentiation and induce CBDC apoptosis (Wang et al., 2012). EBV latent antigen EBNA1 has been anticipated to be involved in BL cell proliferation and resistance to apoptosis, therefore, conferring a selective benefit to malignant cells (Fuentes-Gonzalez et al., 2013). Also, EBNA1 was found to have an anti-apoptotic effect in BL cells (Kutok and Wang, 2006). Several studies suggested that EBNA1 is sufficient to support the neoplastic growth of BL cells *in vivo*, independent of any other latent EBV antigens (Yamamoto et al., 2000). Moreover, studies by Kennedy et al. (2003) demonstrated that overexpression of EBNA1 mutants reduced cell survival and increased apoptosis in EBV-positive BL cells but not in EBV-negative B cells. Interestingly, their work suggested that EBNA1 is important for suppressing cell death in microenvironments (Kennedy et al., 2003). Recent report showed that expression of V-val subtype of EBNA1 in Human Embryonic Kidney cells promotes cell survival after serum withdrawal and provides the anti-apoptotic ability to those cells (Chao et al., 2014). *In vivo* EBNA1 has been confirmed to lower p53 levels (Cheng et al., 2009). Specifically, expression of EBNA1 but not a cellular ubiquitin-specific protease (USP7)-binding mutant of EBNA1 was shown to reduce the accumulation of p53 and apoptosis in response to DNA damage in U2OS cells (Saridakis et al., 2005). Interestingly, transcriptional profiling of Ad/AH carcinoma cells with and without stable EBNA1 expression showed that the presence of EBNA1 resulted in an increased expression of STAT1, a protein that contributes in multiple ways to apoptotic and non-apoptotic cell death (Wood et al., 2007). Other studies suggested that EBNA1 has multiple effects on the oxidative stress response that could affect apoptosis and DNA integrity (Frappier, 2012). Recently, Lu et al. (2010) observed that EBNA1 can contribute to the oncogenic process by up-regulating the apoptosis suppressor protein, survivin in EBV-associated B-lymphoma cells (Lu et al., 2010).

*EBNA2.* EBV nuclear antigen 2 (EBNA2) is the earliest latent-cycle protein of EBV and is essential for B-cell immortalization, proliferation and survival as well as chemotaxis (Fu et al., 2013). Studies by Pegman et al. (2006) demonstrated that in EBV-negative BL-derived cell lines EBNA2 up-regulates bfl-1 expression by interacting with EBNA2-CBF-1. These molecular interactions involve receptors of the classical Notch pathway. EBNA2 also up-regulates other anti-apoptotic proteins such as bfl-1, Bcl-xL, Bcl-2, and MCL-1 and induces the expression of pro-apoptotic proteins including, Bim and Bid (Kohlhof et al., 2009). Recent studies demonstrated that EBNA2 is crucial for suppressing Bik in EBV-negative B-cell lymphoma-derived cell lines and therefore, the host-virus interaction can prevent the pro-apoptotic consequence of transforming growth factor β1 (TGF-β1; Campion et al., 2014). Moreover, EBNA2 was found to inhibit the Sindbis virus (SV)-induced apoptotic response through the interaction with an orphan member of the nuclear hormone receptor superfamily, Nur77 (Lee et al., 2002).

*EBNA3A.* Genetic studies have revealed that both EBNA3A and EBNA3C are responsible for efficient immortalization in EBV infected B cells (Tomkinson et al., 1993). Studies by Cooper et al. (2003) indicated that EBNA3A overexpression impedes protection from c-myc-induced apoptosis in lymphoblastoid cells. Recombinant strategies to delete EBNA3 genes and the infection of EBV-negative BL cells with these viruses and challenging them with various cytotoxic drugs demonstrated that both EBNA3A and EBNA3C cooperate for both drug resistance and the down-regulation of the pro-apoptotic Bcl-2-family member Bim. The regulation of Bim was observed predominantly at the RNA level, with little evidence of post-translational stabilization of Bim by EBV (Anderton et al., 2008). Several evidences strongly suggested that EBNA3A and EBNA3C together inhibit the initiation of BIM transcripts (Paschos et al., 2012). Previous study has also shown that heritable epigenetic modifications initiated by EBNA3A and EBNA3C in the 5′ regulator region of BIM play a vital role in determining the level of post-transcriptional BIM production expressed in EBV-infected B cells (Paschos et al., 2009).

*EBNA3B.* The co-activation activities of EBNA-3A and EBNA-3B are found to be around the half of EBNA3C (Lin et al., 2002). Although EBNA-3B is dispensable for B-cell transformation, both EBNA3A and EBNA-3C are essential (Chen et al., 2005). Among six latency-associated EBNAs, only EBNA3B is completely dispensable for B-cell transformation *in vitro* and could be a tumor suppressor (White et al., 2012). In contrast to EBNA-3A and EBNA3C, both of which repress transcriptions of tumor suppressors, p14ARF, p16INK4A, and chemokine, CXCL10, EBNA-3B upregulates CXCL10 and has a growth inhibitory role (Kang and Kieff, 2015). Importantly, EBNA-3B-mutated B-cell lymphomas were frequently found and evident that EBNA-3B inactivation drives lymphomagenesis and immune evasion (White et al., 2012).

*EBNA3C.* Functional p53 and its downstream effectors are deregulated by several viral antigens to protect host cells from p53-dependent apoptosis during cancer progression (Saha et al., 2010b) EBNA3C was observed to have potential inhibitory effects on p53-mediated activities (Yi et al., 2009). Several studies have shown that EBNA-3C can physically interact with p53 via the specific region, 130–190 amino acid residues in the N-terminal domain which has also been shown to interact with several other important cellular factors, including SCFSkp2, pRb, c-Myc, cyclin A, cyclin E, cyclin D1, and RBP-Jκ (Knight et al., 2004, 2005; Maruo et al., 2009; Saha et al., 2009, 2011). Studies have demonstrated that EBNA3C recruits MDM2 E3-ubiquitin ligase activity for augmenting proteasome dependent degradation of p53 (Saha et al., 2009). Also, EBNA3C can form a p53-independent stable complex with both ING4 and ING5 in EBV-transformed LCLs (Saha et al., 2010a). Recently, it has been reported that EBNA3C strongly binds and stabilizes ATP-dependent RNA helicase DDX20 or Gemin3 expression in EBV-transformed cells (Cai et al., 2011). As a result of EBNA3C-Gemin3 interaction, Gemin3 was found to form a complex with p53 and this is crucial for inhibiting p53-dependent transcriptional activity and apoptosis (Cai et al., 2011). Interestingly, EBNA3C expression was observed to abrogate p73-mediated apoptotic response in p53-null cells (Sahu et al., 2013). Recent studies by Saha et al. (2012) demonstrated that EBNA-3C could directly regulate E2F1 functions to modulate both, cell cycle and apoptotic activities in EBV-transformed B-lymphoma cells. Moreover, EBNA3C was found to interact and differentially regulate the functions of Interferon regulatory factors 4 and 8 in lymphoblastoid cells (LCLs) for apoptotic inhibition (Banerjee et al., 2013). In addition, EBNA3C regulates apoptosis by altering the signaling of several cellular kinases including, Pim-1, Aurora kinase-B (AK-B; Jha et al., 2013a; Banerjee et al., 2014).

*EBNA-LP.* EBNA-LP is considered as a critical regulator of EBV-induced B-cell immortalization, based on the studies that demonstrated less efficiency in the phenotype for recombinant EBNA-LP mutant viruses (Mannick et al., 1991). EBNA-LP has also been observed to interact with several cellular proteins, including oncogenes and tumor suppressors (pRb, p53, p14ARF, and Fte1/S3a), heat shock proteins (hsp70 and hsp72/hsc73), cell-cycle regulatory molecules (DNA-PKcs and HA95) and anti-apoptotic (HAX-1) protein (Szekely et al., 1993; Mannick et al., 1995; Kawaguchi et al., 2000; Dufva et al., 2001; Han et al., 2001; Kashuba et al., 2005). Several studies suggested that truncated form of the EBNA-LP protects some BL cells against caspase-dependent apoptosis by impeding the functions of protein phosphatase 2A (Garibal et al., 2007). In a study by Kawaguchi et al. (2000) using the yeast two-hybrid system, EBNA-LP was found to interact with HAX-1 and it is plausible that EBNA-LP affects the activities of HAX-1 in the regulation of apoptosis during the EBV-induced immortalization process.

*LMP1.* LMP1 not only up-regulates anti-apoptotic proteins to provide support for viral replication but also potentiates apoptosis (Zhang and Huang, 2009). LMP1 stimulates pro-apoptotic gene PAC1 as well as anti-apoptotic genes such as Bcl-2A1/Bfl-1 and A20 (Dirmeier et al., 2005). LMP1 mediated induction of pro-apoptotic genes are likely to be involved in lymphocyte proliferation (Tibbetts et al., 2003). Studies have suggested that the immortalization effect of LMP-1 on B-lymphomas is mediated by the Bcl-2 through a possible cooperation between Bcl-2 and MCL-1. Lu et al. (1997) suggested that LMP-1-induced apoptosis is specifically blocked by the abnormal expression of Bcl-2 or co-expression of LMP-1 and Bcl-2 in epithelial cells (RHEK-1 cells). Down-regulation of Bcl-2 was observed by direct silencing of LMP-1 in an EBV-transformed B-cell line (Noguchi et al., 2001). Several studies also suggested the role for LMP-1 to induce the expression of Bcl-2 in BL cell lines *in vitro* (Finke et al., 1992). Moreover, a positive link between LMP-1 and Bcl-2 has been observed in acquired immune deficiency syndrome (AIDS)-related primary brain lymphomas *in vivo* as well as in NPC (Camilleri-Broet et al., 1995). Other studies were strongly supported by the findings that the expression of bfl-1 suppresses apoptosis stimulated by the amino-terminal six-transmembrane domain (6TM) of LMP-1 (Pratt et al., 2012). Studies by Kim J.H. et al. (2012) also demonstrated that up-regulation of MCL-1 by LMP-1 promotes survival of rituximab-treated B-cell lymphoma cells. Interestingly, the down-regulation of MCL-1 expression is inhibited by LMP-1 in response to apoptotic stimulation (Fu et al., 2013). Interestingly, overexpression of wild type LMP1 was found to be associated with a significant increase in CD95-mediated apoptosis (Le Clorennec et al., 2006). Moreover, several studies suggested that the ability of LMP1 to activate NF-κB was responsible for inducing A20 zinc finger protein to give protection from the cytotoxic effects of TNF-α (Young et al., 1997). Studies by Lu et al. (1996) indicated that higher level of LMP1 expression was responsible for inducing growth arrest and apoptosis for rodent cell transformation.

*LMP2A.* EBV latent membrane protein 2A (LMP2A) was identified in germinal center B cells (Babcock et al., 2000), but its transcripts were detected in all forms of EBV latency, including, resting memory B cells, infectious mononucleosis, Hodgkin lymphoma, BL, and post-transplant lymphoproliferative disorder (PTLD; Thorley-Lawson and Gross, 2004). Therefore, LMP2A was considered crucial in EBV-associated diseases and studies have demonstrated that it has a critical function to rescue cells from apoptosis by potentially altering the balance of pro-apoptotic and pro-survival Bcl2 family members, particularly by mediating the expression of Bcl-xL and Bcl-2 (Swanson-Mungerson et al., 2010). Importantly, both the PI3K/Akt and the Raf/ERK MEK/ERK signaling increases NF-κB, which is a critical mediator of Bcl-xL (Steelman et al., 2004). It has been reported that LMP2A possibly sustains cell survival by modifying Bcl-xL and Bcl-2 expression levels in absence of B-cell receptor signaling (Portis and Longnecker, 2004). Other studies also have demonstrated that LMP2A can bypass the entire p53 pathway in lymphomagenesis involving c-MYC (Bieging et al., 2009).

*EBERs.* The two EBV-encoded small RNAs (EBERs) were identified as EBER-1 and EBER-2. They are small nuclear RNAs transcribed by RNA polymerase III and are the most abundantly expressing EBV transcripts (Rymo, 1979). Several studies have shown that EBV-mediated inhibition of apoptosis and up-regulation of the Bcl-2 protein are essential for the malignant phenotype (Marin et al., 1995). Moreover, previous reports also provided direct evidence that EBV induces Bcl-2 expression by blocking the activation of the double-stranded RNA-dependent protein kinase (PKR; Komano et al., 1999). Interestingly, studies by Wong et al. (2005) suggested that EBER-induced up-regulation of Bcl-2 expression leads to an inactivation of PKR and inhibition of p38 MAPK and C-jun phosphorylation. Additionally, EBER expression may confer an apoptotic-resistant phenotype in immortalized nasopharyngeal epithelial cells (Wong et al., 2005).

*BARTs.* EBV was the first human virus in which the expression of miRNAs, such as MIR-BamHI A rightward transcripts 5 (BART5) was identified (Pfeffer et al., 2004). Unlike cellular miRNAs (Grundhoff et al., 2006), the roles of most EBV miRNAs are well documented. Previous studies suggested that EBV miRNAs are central mediators of viral gene expressions (Yin et al., 2008), however, recent studies demonstrated that MIR-BART5 promotes host cell survival by targeting PUMA expression and contributes to the establishment of latent infection in NPC and germinal center B-cells (Choy et al., 2008). MIR-BARTs may be important in epithelial cells carcinogenesis as they are abundantly expressed in latently infected epithelial cells as compared to the B-cells (Cai et al., 2006). Recent study also suggested the role of EBV-BART microRNAs in targeting the pro-apoptotic protein, Bim (Marquitz et al., 2011). Most of the viral miRNAs belong to the herpesviruses, including human α-herpesviruses such as herpes simplex virus 1 (HSV-1) and HSV-2, avian α-herpesviruses MDV1 (Marek’s disease virus type 1) and MDV2 (Marek’s disease virus type 2), β-herpesvirus human cytomegalovirus (HCMV), and γ-herpesviruses EBV and KSHV (Gottwein and Cullen, 2008). Interestingly, recent studies by Xu et al. (2011) demonstrated that MDV1 microRNA miR-M3 suppresses cisplatin-induced apoptosis by targeting SMAD2 of the TGF-β Signal Pathway.

#### Rhadinoviruses

##### Kaposi’s sarcoma-associated herpesvirus (KSHV)

KSHV was discovered in 1994 from the AIDS-associated KS (Kaposi’s sarcoma) lesions (Chang et al., 1994). KSHV is detected in all cases of KS that develop in HIV-infected as well as HIV-negative individuals. In addition, KSHV sequences are rapidly identified in two other lymphoproliferative and neoplastic disorders: B-cell lymphoma called primary effusion lymphoma (PEL), and the a plasmablastic variant of Multicentric Castleman’s disease (MCD), which contains large plasmablastic cells characterized by the expanded germinal centers with B-cell proliferation and vascularization. KSHV is also associated with several acute inflammatory syndromes (Ganem, 2006). There is also a report of KSHV-linked germinotropic lymphoproliferative disorder in HIV-seronegative individual (Du et al., 2002)

##### Role of KSHV antigens in modulating apoptotic signaling for cancer progression

*LANA.* KSHV LANA (Latency-associated nuclear antigen) encoded by ORF73 is KSHV’s major latency protein and is constitutively active in KS, MCD, and PEL cells. The multifunctional nuclear phosphoprotein, LANA is crucial for KSHV genome maintenance and segregation and plays a key role in regulating several cellular pathways critical for oncogenesis. In addition to being guardian of KSHV latency, LANA binds to and inhibits the cell cycle checkpoint protein and tumor suppressor, p53 as well as transforms primary rat embryo fibroblast (Friborg et al., 1999; Radkov et al., 2000; Borah et al., 2004; Si and Robertson, 2006; Liu et al., 2007). Moreover, LANA interacts with G1-S checkpoint proteins, pRB and GSK-3 (glycogen synthase kinase 3), a negative regulator of β-catenin and modulates G1-S transition (Fujimuro et al., 2003). LANA prolongs the life span of primary human umbilical vein endothelial cells in culture and makes them less susceptible to apoptosis (Watanabe et al., 2003). Like many other cellular proteins, LANA binds to the phosphorylated DNA-damage response protein, γH2AX and the cellular replication fork factors, Timeless and Tipin for LANA-mediated KSHV episome persistence (Dheekollu and Lieberman, 2011; Dheekollu et al., 2013; Jha et al., 2013b). It also associates with different host cellular proteins involved in transcriptional regulation, such as CBP, RING3, activating transcription factor-4/cyclic AMP response element binding protein-2 and mSin3A (Platt et al., 1999; Krithivas et al., 2000; Lim et al., 2001; Verma et al., 2007). These associations have anti-apoptotic and anti-proliferative effects in various KSHV-infected cell lines. A recent study showed that LANA promotes the induction of chromosomal instability through its interaction with Bub1, one of the important spindle checkpoint proteins (Sun et al., 2014). Additionally, LANA was found to dysregulate Bub1 activity leading to aberrant chromosome replication, thus promoting oncogenesis (Sun et al., 2014). A 2014 study by Lu et al. (2014) demonstrated LANA through the interaction with AK-B, can induce phosphorylation of survivin at T34 residue to promote KSHV latent DNA replication and prevent apoptosis. LANA also helps in evading the host’s immune surveillance system and allows the virus to persist indefinitely in the infected host (reviewed in Zaldumbide et al., 2007; Kwun et al., 2011; Uppal et al., 2014). LANA specifically blocks CIITA expression by suppressing the IRF-4-mediated transcription to disrupt the expression of MHC II (Cai et al., 2013). Additionally, LANA down-regulates MHC II expression by disrupting enhanceosome assembly through its binding with the RFX (Regulatory Factor X) Complex (Thakker et al., 2015).

*v-Cyclin.* KSHV ORF72 encoded *v-*Cyclin, expressed during viral latency, is the viral homolog of cellular D-type cyclins (Li et al., 1997). It contributes to the abnormal characteristics of KS spindle cells and proliferation in PEL cells. Interestingly, expression of the KSHV *v-*Cyclin induces cellular apoptosis and B-cell lymphomas in p53-deficient transgenic mice (Verschuren et al., 2002). KSHV *v*-Cyclin is expressed along with KSHV vFLIP from a bicistronic mRNA and silencing of either of these KSHV latent protein by shRNA/siRNA has been shown to induce apoptosis in PEL cells (Guasparri et al., 2004). *v*-Cyclin shares sequence and functional homology with cellular cyclin D2 and can bind and activate the cyclin-dependent kinases, namely CDK6, CDK4, and CDK2 (Godden-Kent et al., 1997; Radkov et al., 2000; Hume and Kalejta, 2009; Pekkonen et al., 2014). When in complex with CDKs, *v-*Cyclin is able to phosphorylate and inactivate many substrates linked to CDKs, including, tumor suppressor protein pRb, cdk inhibitor p27 (Kip) and the anti-apoptotic protein Bcl-2, thereby deregulating normal cell cycle progression (Godden-Kent et al., 1997; Ellis et al., 1999; Ojala et al., 2000). The *v*-cyclin/CDKs complexes are insensitive to CDK inhibitors such as p16INK4a, p21CIP1, and p27KIP1, and can stimulate the cell cycle progression into S-phase (Jarviluoma et al., 2004). In contrast, the expression of *v*-Cyclin in cells with elevated levels of CDK6 triggers cell death independent of p53 and pRb, after the cells enter into S-phase (Ojala et al., 1999). These evidences indicate that *v*-Cyclin is likely to have both growth stimulating and apoptotic functions in KS tumorigenesis.

*vFLIP.* KSHV ORF K13 encoded and latency-associated *v*FLIP *or* FADD-like interleukin-1-beta-converting enzyme (FLICE or caspase-8)-inhibitory proteins, is a viral homolog of cellular FLIP (Thome et al., 1997). This viral protein from KSHV is structurally related to death effector domain (DED) and can bind to the adaptor proteins (TRADD and FADD) of the Fas/TNFR signaling pathway *via* their two tandem DEDs to inhibit CD95-death receptor-induced apoptosis (Thome et al., 1997). Several studies suggested that *v*FLIP induces anti-apoptotic transcription factor NF-κB *via* binding to IKK α, IKK β, RIP, and the NEMO complex (Liu et al., 2002; Matta et al., 2003). In support of this finding, another study showed that KSHV *v*FLIP induction of NF-κB activity impairs autophagosome elongation in latently infected cells (Lee et al., 2009). This showed that the expression of cellular and viral FLIP (cFLIP and KSHV *v*FLIP) suppresses starvation or rapamycin-induced autophagic cell death of KSHV infected B-lymphocytes, by preventing E3-like enzyme, ATG3 from binding and processing LC3. These reports confirm that KSHV *v*FLIP serves as both, an anti-apoptotic as well as an anti-autophagic viral protein, which is essential for the survival and transformation of infected cells (Lee et al., 2009). KSHV *v*FLIP oncoprotein also induces B-cell trans-differentiation and potentially contributes to immune dysfunction during tumor development in mice (Ballon et al., 2011).

*Kaposin.* KSHV ORF K12 encoded Kaposin, is a latent oncogenic protein with potential to transform cells in nude mice and in a fibroblast-transformation assay (Muralidhar et al., 1998; Kliche et al., 2001). There are three isoforms of Kaposin, named as A, B, and C (Sadler et al., 1999). An earlier study showed that Kaposin A, the smallest isoform, directly interacts with cytohesin-1, a guanine nucleotide exchange factor for ARF GTPases, to regulate integrin-mediated cellular transformation and activation of the ERK/MAPK pathway (Kliche et al., 2001). Kaposin B enhances the stabilization of PROX1 mRNA, the master regulator of lymphatic endothelial cell differentiation, during lymphatic reprogramming of vascular endothelial cells by KSHV (Yoo et al., 2010).

*vBcl-2.* KSHV ORF16 encodes protein *v*Bcl-2 with homology to cellular anti-apoptotic protein, Bcl-2, which is characterized by its ability to modulate cell death by dimerizing with other Bcl-2 family members, such as Bax and Bak (Cheng et al., 1997). The *v*Bcl-2 protein is expressed as an early gene during lytic replication and has been shown to inhibit apoptosis to promote viral life cycle through the inhibition of pro-apoptotic BH3 domain-containing proteins (Sun et al., 1999; Flanagan and Letai, 2008). In addition to apoptosis, several studies indicate that vBcl-2 contributes to immune evasion in all gammaherpesviruses *via* inhibition of autophagy (Polster et al., 2004). Contrary to the cellular counterpart, vBcl-2-mediated inhibition of autophagy involves direct and robust interaction of vBcl-2 protein with host Beclin I, the main target of vBcl-2 proteins of KSHV during chronic infection (Liang et al., 2015). Interestingly, Beclin I-mediated inhibition of autophagy and suppression of apoptosis by vBcl-2 are considered as important mechanisms that might contribute to persistent latent infection and the oncogenic potential of KSHV.

*vIRFs.* KSHV encodes four *v*IRFs, namely, *v*IRF-1, *v*IRF-2, *v*IRF-3, and *v*IRF-4, that are homologs of the cellular IRF proteins [interferon (IFN)-regulatory factors], a large family of cellular transcription factors that drive the expression of type I IFNs (IFNα and β), which are produced in nearly all cell types to trigger cell’s innate responses to virus infection by establishing the “anti-viral state” (reviewed in Jacobs and Damania, 2011). They are also known to play a key role in the modulation of cell growth, differentiation and cell death. All KSHV-*v*IRFs have been independently identified to subvert cell cycle arrest by inhibiting p53-mediated apoptosis, either by targeting p53 itself or by targeting its modulators, such as MDM2, HAUSP, and ATM, a function that could potentiate *v*IRF-mediated oncogenesis (Baresova et al., 2013). Of the four KSHV *v*IRFs, only *v*IRF-1, *v*IRF-2, and *v*IRF-3, are shown to effectively inhibit both IFN production and signaling in the infected cells (Baresova et al., 2013). Importantly, both *v*IRF-1 and *v*IRF-3 inhibit p53-induced apoptosis by interacting with the central DNA-binding domain (DBD) of p53 and ATM kinases, and greatly reduce the levels of p53 phosphorylation on serine residues S15 (Shin et al., 2006; Baresova et al., 2014). This results in an increased p53 ubiquitination by MDM2 that predisposes p53 toward proteasome-mediated degradation. Also, *v*IRF-1 inhibits the transforming growth factor-beta (TGF-β) signaling through its targeting of Smad 3 and Smad 4 proteins (Seo et al., 2005). The *v*IRF-3 has been identified as an oncogene required for proliferation and survival of KSHV-infected cultured PEL cells (Wies et al., 2008). The silencing of *v*IRF-3 expression by various RNAi approaches resulted in reduced proliferation and increased activity of caspase-3 and/or caspase-7. In a recent study, *v*IRF-4 but none of the other *v*IRFs, was shown to interact with CSL/CBF1 signaling, the major downstream effector of the Notch signal transduction pathway (Baresova et al., 2013). Moreover, *v*IRF-1 and *v*IRF-2 act as modulators of the immune system by repressing activation-induced cell death (AICD) via modulation of TCR/CD3-mediated induction of CD95L (Chow et al., 2000). Additionally, *v*IRF1 has similar DNA-binding domains to IRF-1 and interacts with p300/CREB-binding protein (CBP) transcription complex, that is required for IRF1- and IRF3-mediated transcription of type I IFNs (Burysek et al., 1999a). The *v*IRF-2 is shown to interact with cellular IRF-1, 2, and 8 as well as NFκB RelA and p300 (Burysek et al., 1999b). The *v*IRF-3 interacts with cellular IRF-5 and inhibits IRF-5-mediated activation of IFN promoter (Wies et al., 2009). Recently, *v*IRFs 1 and 2, but not *v*IRF3, have been reported to suppress endogenous IFNβ message and protein expression following TLR3 activation (Jacobs et al., 2013).

*vMIP.* KSHV ORK K6, ORF K4, and ORF K4.1 encodes for three chemokines or macrophage-inhibitory proteins (*v*MIPs or *v*CCLs), homologous to cellular chemokines/MIPs: viral CC-chemokine ligand-1 (*v*CCL-1 or *v*MIP-1), ligand-2 (*v*CCL-2/*v*MIP-2) and ligand-3 (*v*CCL-3/*v*MIP-3), respectively (Nicholas et al., 1997). In fact, *v*MIP-1 and *v*MIP-2 are more homologous to one another than with cellular MIPs, indicating a gene duplication event during the virus evolution (Moore et al., 1996). Interestingly, *v*MIP-1 is a ligand and agonist of host CC-chemokine receptor (CCR8; Endres et al., 1999), whereas *v*MIP-3 is shown to be specific agonist for host CCR4 (Stine et al., 2000). In addition, *v*MIP-3, when expressed in KS lesions, stimulates angiogenesis, and selectively chemo attracts TH2-type T cells, indicating an important role of *v*MIP-3 in the pathobiology of KS (Stine et al., 2000). The receptors targeted by *v*MIP-2 indicate evasion from a cytotoxic immune response via Th2 polarization and blocking of leukocyte trafficking.

*K1.* KSHV K1 is a transmembrane glycoprotein encoded by the first ORF of the KSHV genome. Initial characterization of K1 protein indicated an early lytic gene expression pattern and identified a highly conserved and functional immunoreceptor tyrosine-based activation motif (ITAM) on the short cytoplasmic tail at its C terminus (Lee et al., 1998a, 2003; Lagunoff et al., 1999). In B lymphocytes, the phosphorylation of ITAMs by protein tyrosine kinases is shown to activate various cellular signal transduction proteins carrying Src homology 2 (SH2) domains, such as PI-3K (p85)/Akt/mTOR, PLCγ_2_, Syk, Cbl, Vav, Lyn, RasGAP (Tomlinson and Damania, 2004; Lee et al., 2005; Prakash et al., 2005), and to induce NFκB, nuclear factor of activated T cells (NFAT), Oct-2 and AP-1 (Prakash et al., 2005). Consequently, K1 expression inhibits proapoptotic proteins and increases the longevity of KSHV-infected cells. The activation of these ITAM-based signal transduction events also contributes to the oncogenic potential of K1 as suggested by tumor formation in mice by K1-transformed rodent fibroblasts and K1-transgenic mice (Lee et al., 1998b). In addition, K1 activation of Akt leads to inactivation of proapoptotic forkhead (FKHR) transcription factor family that protects cells from FKHR- and Fas-mediated apoptosis (Tomlinson and Damania, 2004). KSHV K1 protein is reported to immortalize and extend the life span of endothelial cells in culture (Wang et al., 2006). The expression of K1 in endothelial cells results in the up-regulation of secreted VEGF and MMP-9. In a recent study, Wen and Damania (2010) identified Hsp90 and ER-associated Hsp40/Erdj3 as cellular binding partners of K1, essential for its anti-apoptotic potential.

*K15.* The gene encoding KSHV K15, a putative integral transmembrane protein, is positioned at the 3’ end of the KSHV genome (Choi et al., 2000). Two highly divergent forms of K15 have been identified: the predominant (P) and minor (M) forms (Poole et al., 1999). K15 is weakly expressed in latently infected PEL cells, but is robustly induced on lytic reactivation with chemical inducers such as phorbol esters (Choi et al., 2000). K15 isolates have a complex splicing pattern and yield multiple K15 proteins containing 4–12 transmembrane spanning domains and a short cytoplasmic domain (Glenn et al., 1999). The short cytoplasmic domain of K15 contains potential SH2- and SH3-binding motifs, a YASIL sequence (necessary for the activation of NF-κB and Ras/MAPK signaling pathways) and binding sites for Src family cellular tyrosine kinases and TRAFs 1, 2, and 3 (Glenn et al., 1999; Brinkmann et al., 2007). K15 is capable of initiating several cellular signal transduction pathways, such as Ras/MAPK, JNK/SAPK, and NF-κB (Brinkmann et al., 2003, 2007; Cho et al., 2008) as well as the NFAT/AP1 transcription factors (Cho et al., 2008). K15 also induces the expression of multiple cellular cytokines and chemokines including IL6, IL8, CCL20, CCL2, CXCL3, IL-1α/β, and Cox2 (Brinkmann et al., 2007; Wang et al., 2007). Studies by Sharp et al. (2002) identified cellular HAX-1 (HS associated protein X-1), an anti-apoptotic protein shown to inhibit Bax-induced apoptosis, as a binding partner to the C terminus of K15, both *in vivo* and *in vitro*, inferring K15 may play a role in maintaining latency and/or preventing apoptosis (Wong and Damania, 2006). Like EBV LMP2A, the expression of K15 and K1 led to the survival of BCR-negative human B cells prone to apoptosis (Steinbruck et al., 2015).

*RTA.* KSHV RTA (Replication and Transcription Activator), encoded by ORF50, functions as the master regulator of the transition from latent-to-lytic replication (Sun et al., 1998, 1999). RTA plays a pivotal role as both an initiator and regulator of KSHV lytic DNA replication as the genetic mutation of RTA leads to impaired lytic reactivation and DNA replication (Sun et al., 1998; Xu et al., 2005). KSHV RTA autoactivates its own promoter and transactivates other important lytic genes, namely vIL-6 (Deng et al., 2002; Bu et al., 2008), polyadenylated nuclear RNA (PAN RNA, reviewed in Rossetto and Pari, 2014), ORF57 (MTA; Lukac et al., 2001; Byun et al., 2002), K-bZIP (Lukac et al., 2001), vIRF1 (ORF-K9; Ueda et al., 2002), ORF-K1 (Bowser et al., 2006), small viral capsid protein (ORF65), ORF56, SOX (ORF37), vOX, and ORF52, by binding to the lytic gene promoters containing RTA-response element (Ueda et al., 2002; Song et al., 2003; Fritz et al., 2006). KSHV LANA is also known to repress lytic reactivation and RTA-mediated autoactivation (Lan et al., 2004, 2005). Studies by Nishimura et al. (2003) found that KSHV RTA induced caspase activation and cell death by apoptosis in uninfected cells but not in infected cells. These results suggested that RTA is an apoptosis inducer that is blocked by an anti-apoptotic pathway in KSHV-infected cells (Nishimura et al., 2003). A study by Gao et al. (2011) reported that the up-regulation of the cellular anti-apoptotic Bcl-2 protein by RTA through its binding with CCN_9_GG-like RTA resonsive elements (RREs)/motifs promotes lytic reactivation and enhanced virion production. These results indicate the existence of a distinct, apoptosis-triggered, accelerated RTA-independent replication pathway with clinical significance for the treatment of KSHV-associated neoplasms (Gao et al., 2011). Interestingly, RTA encodes an ubiquitin E3 ligase activity that targets multiple cellular and viral proteins, such as IRF-7, a critical mediator of type I IFN induction, for proteasome-mediated degradation (Yang et al., 2008). Since, IFN signaling plays a critical role in suppressing viral lytic replication, this finding suggests that RTA may follow an unexpected regulatory strategy for overcoming the host innate immune defenses during KSHV reactivation. Another study reported RTA-mediated degradation of the Hey1 repressor protein through the Ubiquitin Proteasome pathway (Gould et al., 2009). Hey1 degradation disrupts the interaction between Hey1 and the co-repressor mSin3A. Hey1 suppresses RTA expression by direct binding to the RTA promoter. RTA is known to up-regulate its own expression by targeting Hey1 protein for degradation. Taken together, these results strongly suggest that RTA regulates viral lytic replication by promoting protein degradation of several cellular repressors. Additionally, recent studies have identified that RTA displays a SUMO-targeting ubiquitin ligase (STUbL) type activity, and is capable of ubiquitylation of SUMO and SUMO conjugates *in vitro* and *in vivo*. Thus, RTA is an ubiquitin ligase that targets SUMO-containing proteins, such as sumoylated K-bZIP and promyelocytic leukemia (PML) nuclear bodies (Izumiya et al., 2013).

*K-bZIP.* KSHV K-bZIP, also known as lytic replication-associated protein (RAP) is a basic leucine zipper-containing protein that is encoded by KSHV K8 (Lin et al., 1999). KSHV K-bZIP is dispensable for lytic reactivation, however, it is crucial for virus production in KSHV-infected cells (Kato-Noah et al., 2007; Rossetto et al., 2007; Lefort and Flamand, 2009; Wang et al., 2011). K-bZIP physically interacts with and represses RTA-mediated transactivation of viral promoters and RTA autoactivation through its basic domain (aa122–189) and a specific RTA region (aa499–550; Izumiya et al., 2003). K-bZIP has been shown to bind and up-regulate the cellular transcription factor CCAAT/enhancer-binding protein-α (C/EBPα) and p21C1P-1 protein, resulting in G0/G1 cell cycle arrest in lytically induced cells (Wang et al., 2003). K-bZIP also efficiently binds to the PRDIII-I region of the IFN-β promoter and prevents the attachment of activated IRF-3 to the IFN-β promoter sequence, suggestive of antagonizing effects exerted by KSHV on type I IFN pathways (Lefort et al., 2007, 2010). Most interestingly, a recent study identified K-bZIP as a SUMO E3 ligase or SUMO adaptor with specificity towards SUMO-2/3 (Chang et al., 2010). In addition, K-bZIP-mediated SUMO-2/3 specific modification on the KSHV genome post reactivation, are found to negatively regulate lytic gene expression and viral reactivation (Chang et al., 2013).

##### Herpesvirus saimiri (HVS)

*Herpesvirus saimiri* is an oncogenic gammaherpesvirus that establishes persistent and replicative infections in different species of primates and transforms human T cells (reviewed in Fickenscher and Fleckenstein, 2001). HVS causes a non-pathogenic, latent infection in its natural hosts, the squirrel monkey, however, in the New World primates such as the common marmosets, it results in severe and rapidly progressing T-cell lymphomas. Furthermore, HVS leads to a lifelong persistent infection primarily in T-lymphocytes. Like other members of the rhadinovirus family, HVS has pirated a number of cellular genes to regulate cell cycle, evade immune surveillance and to inhibit apoptosis.

##### Role of HVS antigens in cancer progression by modulation of apoptotic signaling

Tip (tyrosine kinase-interacting protein) encoded by HVS subtype C, located downstream of Stp gene, is a transforming protein important for viral transformation. HVS Tip has been reported to induce and immortalize infected human T-cells *in vitro* (Biesinger et al., 1992). Tip has multiple binding sites for cellular proteins. Indeed, Jae Jung’s group has reported that the HVS Tip interaction with p80 and subsequent recruitment of Lck and TCR/CD3 complexes to lipid rafts markedly inhibits the T-cell receptor (TCR)-mediated intracellular signal transduction and CD4 surface expression. Strikingly, these two interactions are reported to be functionally and genetically separable, i.e., the interaction of its N-terminal region with p80 is responsible for TCR down-regulation, whereas, the interaction of its C-terminal domain with Lck governs the CD4 down-regulation (Park et al., 2002, 2003; Cho et al., 2004, 2006). In addition, HVS Tip protein binding to Lck kinase requires SH3 Binding motif (SH3B) and C-terminal Src-related Kinase Homology (CSKH) element of Tip (Jung et al., 1995). Recently, the group also reported that the association of membrane-proximal amphipathic helix with Tip’s transmembrane (TM) domain is sufficient for localization to lipid rafts and deformation of cellular membrane, which in turn directs Tip’s lysosomal trafficking and selective TCR down-regulation (Min et al., 2008). Interactions between the phosphorylated peptides of HVS Tip and the Src homology 2 (SH2) domains of STAT3 and STAT6 facilitate Src kinase-mediated STAT-activation and T-cell proliferation (Mazumder et al., 2012). Another group recently reported that the N-terminal end sequence of Tip associates with and inhibits cellular retromer activity, thus leading to CD4 down-regulation and efficient T-cell transformation in an IL-2 independent fashion (Kingston et al., 2011). Tip can also induce T-cell transformation independent of IL-2 by constitutively activating the STAT6 transcription factor by interacting with and phosphorylating STAT6 (Kim Y. et al., 2012). Tip has been found to activate the serum response element (SRE) in a Lck and Src-family kinase interaction-dependent fashion, indicating its potential role in actin-regulated transcription and transformation of human T cells (Katsch et al., 2012).

*Herpesvirus saimiri* encodes a potent complement inhibitor, a structural homolog of complement control proteins, CCPH that inhibits C4b as well as C3b deposition on the target cells, exposed to complement, thus allowing HVS to evade the host complement attack (Singh et al., 2006; Reza et al., 2013). HVS CCPH effectively inactivates complement by supporting factor I-mediated inactivation of complement proteins, C3b and C4b. In a recent study, Reza et al. (2013) performed substitution mutagenesis of CCPH residues (sCCPH mutants) and demonstrated that ionic charges within amino acids form a major component of binding interface between CCPH and its interacting partners. These charges are reported to be crucial for CCPH’s interaction in human and viral complement regulators. In addition, the HVS genome encodes for one or more viral cytokines to promote survival of the infected cell and escape from the host immune responses. HVS encodes a viral interleukin, IL-17, which has been shown to support T-cell proliferation and uncontrolled cellular growth by up-regulating NF-κB, and IL-6 expression levels (Yao et al., 1995). HVS-transformed T cells are known to display an elevated expression of cellular IL-26, an IL-10-related cytokine that results in STAT1 and STAT3 activation (Hor et al., 2004). Moreover, HVS ORF3 protein, the viral FGARAT-homologous protein, was recently found to induce the proteasomal degradation of the cellular ND10 component Sp100 (Full et al., 2012). A study by Cazalla et al. (2010) showed that viral U-rich non-coding RNAs down-regulate the expression of host miRNA (miR-27), which in turn led to an enhanced IFN-γ levels in transformed T-cells to promote latency (Cazalla et al., 2010). The group later reported that HVS microRNAs, termed as miR-HSURs, preferentially modulate the expression of host cell cycle regulators (WEE1) and antiviral response factors (MHC-1 complex; Guo et al., 2015).

##### Rhesus macaque rhadinovirus (RRV)

Two separate groups identified RRV from rhesus macaques in 1997 (Desrosiers et al., 1997) and 1999 (Wong et al., 1999) at the New England Primate Research Center and Oregon National Primate Research Center, respectively. RRV is a natural pathogen of rhesus macaques (RS) monkey, persists latently in B-lymphocytes, and is known to cause B-cell hyperplasia and persistent lymphadenopathy (Desrosiers et al., 1997; Searles et al., 1999). Genome sequence analysis of RRV 17557, one of the two isolated strains of RRV, revealed a high degree of co-linearity with another rhadinovirus, i.e., KSHV (Searles et al., 1999). The second isolate of RRV, 26–95 also has high degree of similarity with both, KSHV and RRV 17557 (Alexander et al., 2000). Studies focusing on the mechanisms underlying KSHV oncogenesis have been impeded due to the lack of a proper animal model and poor replication of the virus in cell culture. In contrast, RRV grows efficiently in cell culture and produces high titers of virus upon induction, hence serves as an excellent model for studying both the *in vivo* and *in vitro* KSHV infection.

#### Role of RRV Antigens in Cancer Progression by Modulation of Apoptotic Signaling

Damania’s group showed that R1 protein of RRV bears significant similarity to the K1 protein of KSHV and initiates B lymphocyte activation through its signal transducing cytoplasmic domain, thereby formulates its role as an oncoprotein (Damania et al., 1999, 2000b). RRV, upon co-infection with Simian Immunodeficiency Virus (Sadagopan et al., 2007) has been reported to develop lymphoproliferative disorders, similar to those in AIDS patients, co-infected with KSHV (Wong et al., 1999). RRV is also known to possess viral interleukin-6, RvIL-6, which potentially plays a role in RRV pathogenesis by regulating host-virus interactions and possibly enhancing host IL-6 signaling (Kaleeba et al., 1999), hence, assisting in B cell proliferation, both *in vivo* and *in vitro* (Kaleeba et al., 1999; Orzechowska et al., 2008). RRV ORF74 has been reported to induce tumor formation in mice and up-regulates VEGF secretion through activation of Erk signaling, resulting in cellular transformation, similar to that caused by ORF74 of KSHV. This confirms that these two proteins are homologous and RRV ORF74 is likely to contribute to RRV-related malignancies in a similar fashion to the KSHV ORF 74 (Estep et al., 2003). Previous studies have reported the reduction in macrophage activation following expression of the protein RRV vCD200, encoded by RRV ORFR15, *in vitro* (Langlais et al., 2006). In addition, a recent study demonstrated the role of RRV vCD200 in modulation of host immune responses at early times post infection. The RRV vCD200 was found to inhibit excess virus production at early time points in order to promote viral infection. These observations indicate a plausible role of vCD200 in direct inhibition of antigen-presenting cells, through decline in the levels of CD200 receptor on dendritic cells (Estep et al., 2014). Also, RRV encodes for eight vIRF proteins that are viral homologs of the cellular IRFs. These RRV vIRFs were reported to interfere with the transcriptional functions of cellular IRFs and led to a reduction in induction of IFNs post infection. These findings were further supported by the results that demonstrate an increase in the IFN production following deletion of the IRFs (Robinson et al., 2012). According to a recent study, the vIRFs inhibit the IFN gene activation by interacting with CREB binding protein, CBP, a transcriptional co-activator and acetyltransferase (Morin et al., 2015). These observations account for an important role of IRFs in hampering the host immune response against the virus. In addition, RRV ORF71 encodes a cellular homolog for FLIP (FLICE, FADD-like interleukin-1-converting enzyme-inhibitory protein) known as viral FLIP (vFLIP), during the latent phase of the virus. The *v*FLIP protein was found to promote cell survival and inhibit apoptosis in latently infected cells through autophagosome formation. The inhibition in *v*FLIP protein expression resulted in the loss of anti-apoptotic property of the cells and concomitant reduction in autophagy (Ritthipichai et al., 2012).

## Conclusion

In summary, the study of tumor viruses has been instrumental to our present knowledge of multiple neoplastic diseases. It is now clear that several γ-herpesviruses, including, KSHV, EBV, and HVS express a diverse repertoire of viral genes that control the cell’s death machinery and contribute to the viral replication and tumor progression in the infected, immuno-compromised hosts at opportune times. These viruses have developed diverse and complex mechanisms to circumvent host-mediated self-destruction and perturb the cellular control of apoptosis, immune recognition and autophagy to their advantage *via* the expression of unique viral anti-autophagic and anti-apoptotic proteins, viral homologs of host proteins and activation of a plethora of cellular signaling proteins to promote viral replication and lifelong persistence **Table 1**. As deregulation of cell signaling pathways is a defining feature of malignantly transformed cells thus, unraveling these mechanisms will definitely delineate new strategies to prevent tumor growth as well as identify causative novel targets suitable for drug development.

**Table 1:** Gammaherpesviruses related to cancer: Some of the key viral oncoproteins affecting apoptosis to promote cell transformation.

Gammaherpesvirus	Gene name	Protein name	Gene functions/Mechanism
Epstein–Barr virus (EBV)	EBNA1	Epstein–Barr nuclear antigen 1	Inhibits p53-dependent apoptosis, essential for viral gene maintenance
	EBNA2	Epstein–Barr nuclear antigen 2	Nuclear protein, transcription factor
	EBNA3A	Epstein–Barr nuclear antigen 3A	Essential for *in vitro* B-cell immortalization, nuclear protein, transcription factor
	EBNA3B	Epstein–Barr nuclear antigen 3B	Nuclear protein, transcription factor
	EBNA3C	Epstein–Barr nuclear antigen 3C	Essential for *in vitro* B-cell immortalization, nuclear protein, transcription factor
	EBNA-LP	Epstein–Barr nuclear antigen leader protein	Interacts with tumor suppressors and anti-apoptotic proteins
	LMP1	Latent membrane protein 1	Upregulates anti-apoptotic proteins
	LMP2A	Latent membrane protein 2A	Upregulates anti-apoptotic proteins, bypasses p53 pathway
	EBERs	Epstein–Barr virus-encoded small RNAs	Regulates bcl-2 expression
	BARTs	BamHI A rightward transcripts	Targets PUMA expression and promotes host cell survival
Kaposi’s sarcoma-associated herpesvirus (KSHV)	LANA	Latency-associated nuclear antigen	Episomal maintenance protein, establishes and maintains latency, inhibit p53-dependent apoptosis,
	*v*Cyclin	*v*Cyclin protein	Induces p53-dependent apoptosis, promotes cell cycle progression and proliferation
	*v*FLIP	Viral FADD-like-inhibitory proteins	Blocks caspase 8 activation, potent activator of NF-κB
	*Kaposin*	Kaposin protein	Transforming protein, induces the expression of growth factor receptors
	*v*Bcl-2	Viral B-cell lymphoma 2	Inhibits apoptosis
	*v*IRFs	Viral interferon (IFN)-regulatory factors	Cellular IRFs homologs, Inhibitor of IFN1, p53, ReIA, NF-κB and p300
	*v*MIP	Viral macrophage-inhibitory proteins	Binds to chemokine receptors and induces angiogenesis
	K1	K1 protein	Type I transmembrane signaling protein, contains ITAM for activating signaling pathways
	K15	K15 protein	Signal modulator, induces various chemokines and cytokines
	RTA	Replication and Transcription Activator	Potent transactivator, master regulator of lytic cycle
	K-bZIP	E3 SUMO-protein ligase K-bZIP	Co-regulator of RTA, regulates lytic-cycle DNA replication
Herpesvirus saimiri (HVS)	Tip	Tyrosine kinase interacting protein	Signal modulator, transforming protein
	CCPH	Complement control protein homolog	Binds to C3b and C4b, blocks complement activation
	vIL-17	Viral interleukin-17	Anti apoptotic protein, supports T-cell proliferation
	ORF3	ORF3 tegument protein	Viral FGARAT homologous protein, induces proteasomal degradation of cellular Sp100
	HSUR1	Herpesvirus saimiri U-rich RNAs	Down-regulates the expression of cellular miRNAs
	miR-HSURs	Herpesvirus saimiri U-rich microRNAs	Modulate the expression of host cell cycle regulators and antiviral response factors
Rhesus macaque rhadinovirus (RRV)	R1	R1 protein	Activates B-lymphocytes signaling
	vIRFs	Viral interferon (IFN)-regulatory factors	Inhibit IFN signaling
	RvIL-6	Viral interleukin-9	Essential for *in vitro* B-cell proliferation
	vCD200	Viral CD200 homolog	Modulates immune responses
	ORF74	Encodes for G protein-coupled receptor	Up-regulates VEGF secretion through activation of Erk signaling

## Author Contributions

All authors listed, have made substantial, direct and intellectual contribution to the work, and approved it for publication.

## Conflict of Interest Statement

The authors declare that the research was conducted in the absence of any commercial or financial relationships that could be construed as a potential conflict of interest.
